# Serum Phosphate and 1-Year Outcome in Patients With Acute Ischemic Stroke and Transient Ischemic Attack

**DOI:** 10.3389/fneur.2021.652941

**Published:** 2021-04-14

**Authors:** Jun-Fang Zhang, Jing Jing, Xia Meng, Yuesong Pan, Yi-Long Wang, Xing-Quan Zhao, Jin-Xi Lin, Xin-Sheng Han, Bin-Bin Song, Zheng-Chang Jia, Song-Di Wu, Xiao-Fei Chen, Wen-Jun Xue, Craig S. Anderson, Yun-Cheng Wu, Yong-Jun Wang

**Affiliations:** ^1^Department of Neurology, Shanghai General Hospital, Shanghai Jiao Tong University School of Medicine, Shanghai, China; ^2^Department of Neurology, Beijing Tiantan Hospital, Capital Medical University, Beijing, China; ^3^China National Clinical Research Center for Neurological Diseases, Beijing, China; ^4^Center of Stroke, Beijing Institute for Brain Disorders, Beijing, China; ^5^Beijing Key Laboratory of Translational Medicine for Cerebrovascular Disease, Beijing, China; ^6^Kaifeng Central Hospital, Kaifeng, China; ^7^Luoyang Central Hospital, Luoyang, China; ^8^The Second People's Hospital of Jinzhong, Jinzhong, China; ^9^Department of Neurology, The First Hospital of Xi'an, Xi'an, China; ^10^Shanxi Cardiovascular Hospital, Taiyuan, China; ^11^Pingdingshan First People's Hospital, Pingdingshan, China; ^12^The George Institute, University of New South Wales (UNSW), Sydney, NSW, Australia

**Keywords:** serum phosphate, stroke, recurrence, mortality, outcome

## Abstract

**Objective:** To determine the association between serum phosphate level and 1-year clinical outcomes in patients with acute ischemic stroke and transient ischemic attack.

**Methods:** We included 7,353 patients with acute ischemic stroke and transient ischemic attack from the China National Stroke Registry III for analysis. Participants were divided into 4 groups according to serum phosphate quartiles. Composite end point included recurrent stroke, myocardial infarction, other ischemic vascular events, and all-cause mortality. Poor functional outcome is defined as modified Rankin Scale score of 3 to 6. Multivariable Cox regression or logistic regression was used to evaluate the independent association of serum phosphate with 1-year all-cause mortality, recurrent stroke, composite end point and poor functional outcome.

**Results:** The mean age of the included 7,353 patients was 62.5 years, and 68.6% of them were men. Plotting hazard ratios over phosphate levels suggested a U-shaped association especially for recurrent stroke and composite end point, and therefore the third quartile group was set as reference group. Compared with the third quartile of phosphate (1.06–1.20 mmol/L), the adjusted hazard ratios/odds ratios (95% CI) of the lowest quartile (<0.94 mmol/L) were 0.98 (0.67–1.42) for all-cause mortality, 1.31 (1.05–1.64) for stroke recurrence, 1.26 (1.02–1.57) for composite end point, and 1.27 (1.01–1.61) for poor functional outcome, and the adjusted odds ratio of the highest quartile (≥1.2 mmol/L) was 1.40 (1.11–1.77) for poor functional outcome.

**Conclusions:** Serum phosphate may be an independent predictor of stroke recurrence, composite end point and poor functional outcome after ischemic stroke.

## Introduction

Phosphorus plays an important role in multiple biological functions, including cellular signal transduction, mineral metabolism, and energy exchange. Serum phosphorus mainly occurs as inorganic phosphate in human body. The level of serum phosphate is tightly regulated by several pathways including dietary absorption, bone formation, renal excretion, and intracellular stores (1–3). The underlying pathological effects of elevated phosphate on cardiovascular organs include vascular calcification and endothelial dysfunction (4). Higher phosphate level is associated with higher rates of cardiovascular events or cardiovascular disease related mortality in general population (5–8) and individuals with underlying coronary artery disease (9), while low serum phosphate level is associated with hypertension and metabolic syndrome in general population (10–12). Although studies investigating dialysis patients (10, 13) focused on the relationships between hyperphosphatemia, hypophosphatemia and the cardiovascular outcomes in the beginning, more and more studies (5–9, 11, 12) found the relationship even within relatively normal ranges.

Current data about the effects of serum phosphate on clinical outcomes in patients after ischemic stroke is limited. Although one study showed a U-shaped association between phosphate and in-hospital mortality with significantly increased risk among ischemic stroke patients with lower phosphate level (14), another study found no association between phosphate and 3-month functional outcome in patients with acute ischemic stroke (15). To date, no comprehensive study of the association between serum phosphate and long-term clinical outcomes of ischemic stroke patients has been investigated.

In this study, we aimed to determine the association between serum phosphate level and 1-year clinical outcomes including all-cause mortality, recurrent stroke, composite end point, and functional outcome, in patients with acute ischemic stroke and transient ischemic attack (TIA).

## Methods

### Study Population

**Figure 1:**
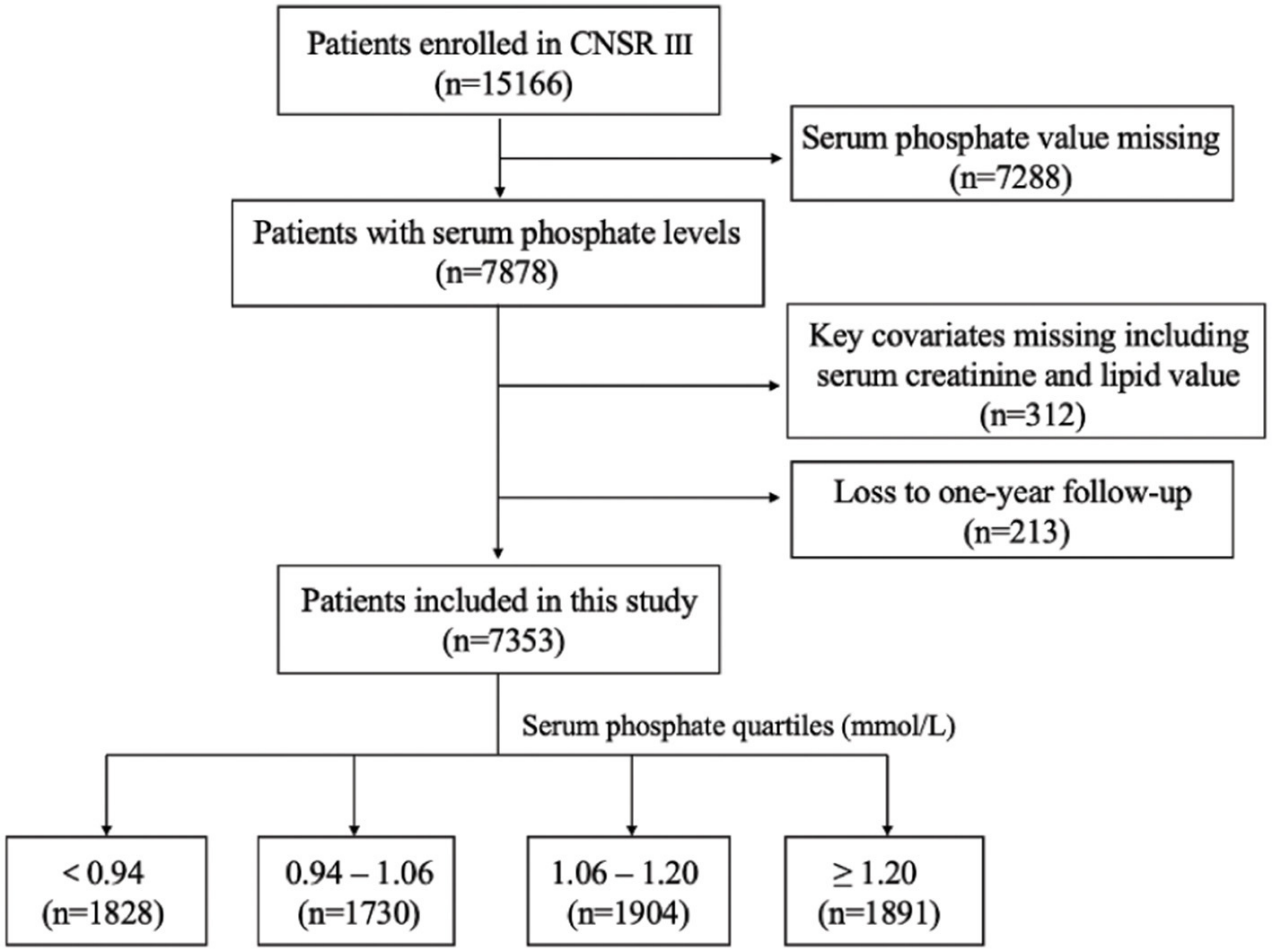
Patient flow diagram. CNSR III, China National Stroke Registry III.

This study was conducted on the basis of the CNSR III study (China National Stroke Registry III), which was a nationwide, hospital-based, prospective cohort study enrolling patients with acute ischemic cerebrovascular events between August 2015 and March 2018 in China. Patients were eligible if they met the following criteria: age 18 years or older; diagnosis within 7 days of the index event of ischemic stroke or TIA. The design, rationale and baseline patient characteristics of CNSR III study have been published previously (16). ^15^ A total of 201 study sites participated in this study with a median number of patients recruited as 55 (minimum, 3; maximum, 321). Among the 15,166 patients in the registry, 7,353 were analyzed after excluding patients with missing serum phosphate value (*n* = 7,288), missing serum creatinine or lipid value for key covariates (*n* = 312) and lost during the 1-year follow-up (*n* = 213; Figure 1).

### Standard Protocol Approvals and Patient Consents

The CNSR III study was approved by ethics committee at Beijing Tiantan Hospital (IRB approval number: KY2015-001-01), and written informed consent was obtained from patients or their legally authorized representatives. The study complied with the principles of the Declaration of Helsinki.

### Baseline Data Collection

Baseline information of included patients, including demographics, vascular risk factors, important laboratory data, treatment and complications were collected by trained research coordinators at each study center. Fasting blood samples were drawn within 24 h of admission and assessed as baseline laboratory data. Risk factors contained stroke history, hypertension, diabetes mellitus, hypercholesterolemia, coronary heart disease, previous or current smoking, and heavy alcohol consumption. Hypertension, diabetes mellitus, and hypercholesterolemia were defined according to (1) documented or self-reported history or (2) receiving medication for corresponding diseases or (3) clinical or laboratory examination (systolic blood pressure ≥140 mmHg or diastolic blood pressure ≥90 mmHg on repeated measurements for a diagnosis of hypertension (17), fasting glucose level ≥126 mg/dl or 2-h plasma glucose ≥200 mg/dl during oral glucose tolerance test (75 g) or random plasma glucose ≥200 mg/dl in persons with symptoms of hyperglycemia or hyperglycemic crisis for diabetes mellitus (18), total cholesterol>240 mg/dl or serum triglyceride ≥200 mg/dl or low-density lipoprotein cholesterol ≥160 mg/dl or high-density lipoprotein cholesterol ≤ 40 mg/dl for dyslipidemia (19)), or (4) new diagnosis at discharge. Heavy alcohol consumption was defined as consuming ≥2 standardized alcohol drinks per day. Estimated glomerular filtration rate (eGFR) was calculated using the Chronic Kidney Disease Epidemiology Collaboration creatinine equation with adjusted coefficient of 1.1 for the Asian population (20, 21).

### Serum Phosphate Testing

Fasting blood samples were drawn within 24 h of admission, and serum phosphate levels were assessed with an ammonium molybdate assay using unfrozen samples in each center. Briefly, the phosphate ions react with ammonium molybdate and then reduced to blue molybdenum which is finally colorimetric measured.

### Clinical Outcome Assessment

Patients were followed up over telephone at 12-month after disease onset by trained research coordinators who were blinded to baseline clinical status. Data on clinical outcomes were collected. We defined adverse clinical outcomes as recurrent stroke, all-cause mortality, and poor functional outcome. Recurrent stroke includes ischemic stroke, intracranial hemorrhage, and subarachnoid hemorrhage. Composite end point was comprised of recurrent stroke, myocardial infarction, other ischemic vascular events, and all-cause mortality. Poor functional outcome is defined as modified Rankin Scale (mRS) score of 3 to 6 [mRS score ranges from 0 (no symptoms) to 6 (death)].

### Statistical Analysis

Proportions were used to describe the categorical variables; means with SD or median with the interquartile range (IQR) were used for continuous variables. We used χ2 test for categorical variables; 1-way analysis of variance or Kruskal-Wallis test were adopted for continuous variables. Univariate and multivariable Cox regression models were performed to estimate the association between serum phosphate and all-cause mortality, recurrent stroke and composite end point, with third quartile of serum phosphate as reference group (14), while univariate and multivariable logistic regression models were performed to estimate the association between serum phosphate and poor functional outcome. Odds ratios (ORs) or hazard ratios (HRs) with their 95% CIs were reported. Variables included in the multivariable model were selected based on baseline characteristics differences between different quartile groups or based on previous studies. These variables were age, sex, history of stroke, hypertension, diabetes mellitus, hypercholesterolemia, coronary heart disease, current or previous smoking, heavy drinker, body mass index (BMI), National Institutes of Health Stroke Scale (NIHSS) score at admission, hemoglobin, serum calcium, serum potassium, serum albumin, estimated glomerular filtration rate, serum creatinine, total cholesterol, triglycerides, mRS at discharge, antihypertensive drugs, lipid-lowering drugs, hypoglycemia drugs, and pneumonia during hospitalization. In addition, we further explored the pattern of association between serum phosphate levels and risk of stroke outcomes using a logistic regression model with restricted cubic splines for serum phosphate adjusting for covariates with 5 knots (at the 5th, 25th, 50th, 75th and 95th percentiles). Furthermore, C statistics, net reclassification index, and integrated discrimination improvement were used to evaluate the incremental prognostic value of serum phosphate levels beyond conventional risk factors (22). All analyses were conducted with SAS version 9.4 software (SAS institute), and 2-tailed *P*-values of <0.05 were considered to be statistically significant.

## Results

### Baseline Characteristics

Compared to patients excluded (*n* = 7,813), the patients included in this analysis were slightly older and more likely to have diabetes history, to be a heavy drinker, and to have lower mRS score at discharge (Supplementary Table 1). Baseline characteristics of included patients are summarized in Table 1. Of the total 7,353 patients, the mean age was 62.5 years, and 68.6% were men. Patients with higher quartiles of serum phosphate were younger, and there were more females, more patients with history of diabetes mellitus, hypercholesterolemia while less smokers. They also had slightly higher BMI and lower NIHSS score. The levels of serum calcium, serum potassium, serum albumin, eGFR, total cholesterol, low-density lipoprotein cholesterol and triglycerides increased along with the levels of serum phosphate, while the levels of hemoglobin and serum creatinine decreased. History of stroke, hypertension, coronary heart disease, alcohol consumption, and level of high-density lipoprotein cholesterol were not significantly different among the quartiles.

**Table 1:** Baseline characteristics of the patients according to quartiles of serum phosphate level.

**Characteristics**	**Overall (*n* = 7,353)**	**Serum phosphate level, mmol/L**				***P*-value**
		**Q1 (<0.94) (*n* = 1,828)**	**Q2 (0.94-1.06) (*n* = 1,730)**	**Q3 (1.06-1.20) (*n* = 1,904)**	**Q4 (≥1.20) (*n* = 1891)**	
Age (mean ± SD), y	62.5 ± 11.4	64.7 ± 11.2	63.5 ± 11.0	61.6 ± 11.3	60.4 ± 11.6	<0.0001
Male sex, *n* (%)	5046 (68.6)	1488 (81.4)	1278 (73.9)	1244 (65.3)	1036 (54.8)	<0.0001
**Risk factors**, ***n*** **(%)**						
Previous stroke	1577 (21.5)	422 (23.1)	378 (21.9)	404 (21.2)	373 (19.7)	0.09
Hypertension	4647 (63.2)	1147 (62.8)	1090 (63.0)	1197 (62.9)	1213 (64.2)	0.80
Diabetes mellitus	1764 (24.0)	380 (20.8)	397 (23.0)	467 (24.5)	520 (27.5)	<0.0001
Hypercholesterolemia	589 (8.0)	114 (6.2)	139 (8.0)	167 (8.8)	169 (8.9)	0.01
CHD	777 (10.6)	208 (11.4)	188 (10.9)	179 (9.4)	202 (10.7)	0.24
Current or previous smoking	3514 (47.8)	949 (51.9)	840 (48.6)	915 (48.1)	810 (42.8)	<0.0001
Heavy drinker	1084 (14.7)	280 (15.3)	248 (14.3)	294 (15.4)	262 (13.9)	0.45
BMI (mean ± SD)	24.7 ± 3.4	24.4 ± 3.2	24.6 ± 3.3	24.8 ± 3.5	24.9 ± 3.5	<0.0001
NIHSS score at admission, median (IQR)	3 (1-6)	4 (2-7)	3 (1-6)	3 (1-6)	3 (1-6)	<0.0001
**Laboratory parameters, (mean** **±** **SD)**						
Hemoglobin, g/L	141.0 ± 17.1	143.1 ± 16.9	141.1 ± 16.5	140.8 ± 16.5	138.9 ± 18.1	<0.0001
Serum phosphate, mmol/L	1.07 ± 0.21	0.82 ± 0.09	1.00 ± 0.03	1.12 ± 0.04	1.33 ± 0.17	<0.0001
Serum calcium, mmol/L	2.2 ± 0.1	2.2 ± 0.2	2.2 ± 0.1	2.3 ± 0.1	2.3 ± 0.1	<0.0001
Serum potassium, mmol/L	3.9 ± 0.4	3.9 ± 0.4	3.9 ± 0.4	3.9 ± 0.4	4.0 ± 0.4	<0.0001
Serum albumin, g/L	40.5 ± 4.1	40.1 ± 4.2	40.4 ± 4.0	40.7 ± 3.9	40.7 ± 4.4	<0.0001
**Kidney function**						
eGFR, mL/min/1.73 m2	90.7 ± 30.8	89.2 ± 29.7	89.6 ± 28.5	91.6 ± 27.5	92.4 ± 36.5	0.003
Serum creatinine, μmol/L	74.1 ± 28.4	75.7 ± 22.9	75.1 ± 26.0	72.4 ± 24.9	73.4 ± 37.3	0.001
**Lipid status (mean** **±** **SD), mmol/L**						
Total cholesterol	4.3 ± 1.2	4.2 ± 1.2	4.3 ± 1.2	4.4 ± 1.3	4.4 ± 1.3	<0.0001
LDL cholesterol	2.6 ± 1.1	2.5 ± 1.0	2.6 ± 1.0	2.6 ± 1.1	2.7 ± 1.1	0.0001
HDL cholesterol	1.1 ± 0.3	1.1 ± 0.3	1.1 ± 0.3	1.1 ± 0.3	1.1 ± 0.3	0.30
Triglycerides	1.6 ± 1.1	1.5 ± 0.9	1.6 ± 1.0	1.7 ± 1.3	1.8 ± 1.2	<0.0001
**mRS score at discharge**, ***n*** **(%)**						
0–2	5977 (81.6)	1415 (77.7)	1421 (82.4)	1578 (83.2)	1563 (82.9)	<0.0001
3–5	1351 (18.4)	406 (22.3)	304 (17.6)	319 (16.8)	322 (17.1)	
**Medication during hospitalization**, ***n*** **(%)**						
Antihypertensive drugs	3412 (46.4)	846 (46.3)	805 (46.5)	862 (45.3)	899 (47.5)	0.58
Hypoglycemia drugs	1897 (25.8)	399 (21.8)	429 (24.8)	515 (27.1)	554 (29.3)	<0.0001
Lipid-lowering drugs	6964 (94.7)	1734 (94.9)	1638 (94.7)	1797 (94.4)	1795 (94.9)	0.88
Pneumonia during hospitalization	432 (5.9)	143 (7.8)	111 (6.4)	96 (5.0)	82 (4.3)	<0.0001

*BMI, body mass index; CHD, coronary heart disease; eGFR, estimated glomerular filtration rate; HDL, high-density lipoprotein; IQR, interquartile range; LDL, low-density lipoprotein; mRS, modified Rankin Scale score; NIHSS, National Institutes of Health Stroke Scale; Q, Quartiles*.

### One-year Outcomes Among Patients Grouped by Quartiles of Serum Phosphate

The 1-year incidences of clinical outcomes are shown in Table 2. The 1-year rates of recurrent stroke, composite endpoint and poor functional outcome were lowest in third quartile group (*p* = 0.03 for recurrent stroke; *p* = 0.03 for composite end point; *p* < 0.0001 for poor functional outcome). There was no significant difference in all-cause mortality among groups (*p* = 0.06). In the lowest serum phosphate quartile, the incidence rates of outcomes including all-cause mortality, recurrent stroke, composite endpoint, and poor functional outcomes were 4.4, 10.8, 11.6, 17.6%, respectively.

**Table 2:** Rates of 1-year outcomes according to quartiles of serum phosphate level.

		**Serum phosphate level, mmol/L**				
**Outcomes**	**Overall**	**Q1(<0.94)**	**Q2(0.94-1.06)**	**Q3(1.06-1.20)**	**Q4(≥1.20)**	***P*-value**
All-cause mortality, *n* (%)	266 (3.6)	81 (4.4)	51 (3.0)	60 (3.1)	74 (3.9)	0.06
Recurrent stroke, *n* (%)	674 (9.2)	197 (10.8)	155 (9.0)	154 (8.1)	168 (8.9)	0.03
Composite end point, *n* (%)	723 (9.8)	212 (11.6)	164 (9.5)	168 (8.8)	179 (9.5)	0.03
Poor functional outcome, *n* (%)	1087 (14.8)	321 (17.6)	261 (15.1)	225 (11.8)	280 (14.8)	<0.0001

*Q, quartiles*.

### Association of Serum Phosphate Levels With Adverse Clinical Outcomes

**Figure 2:**
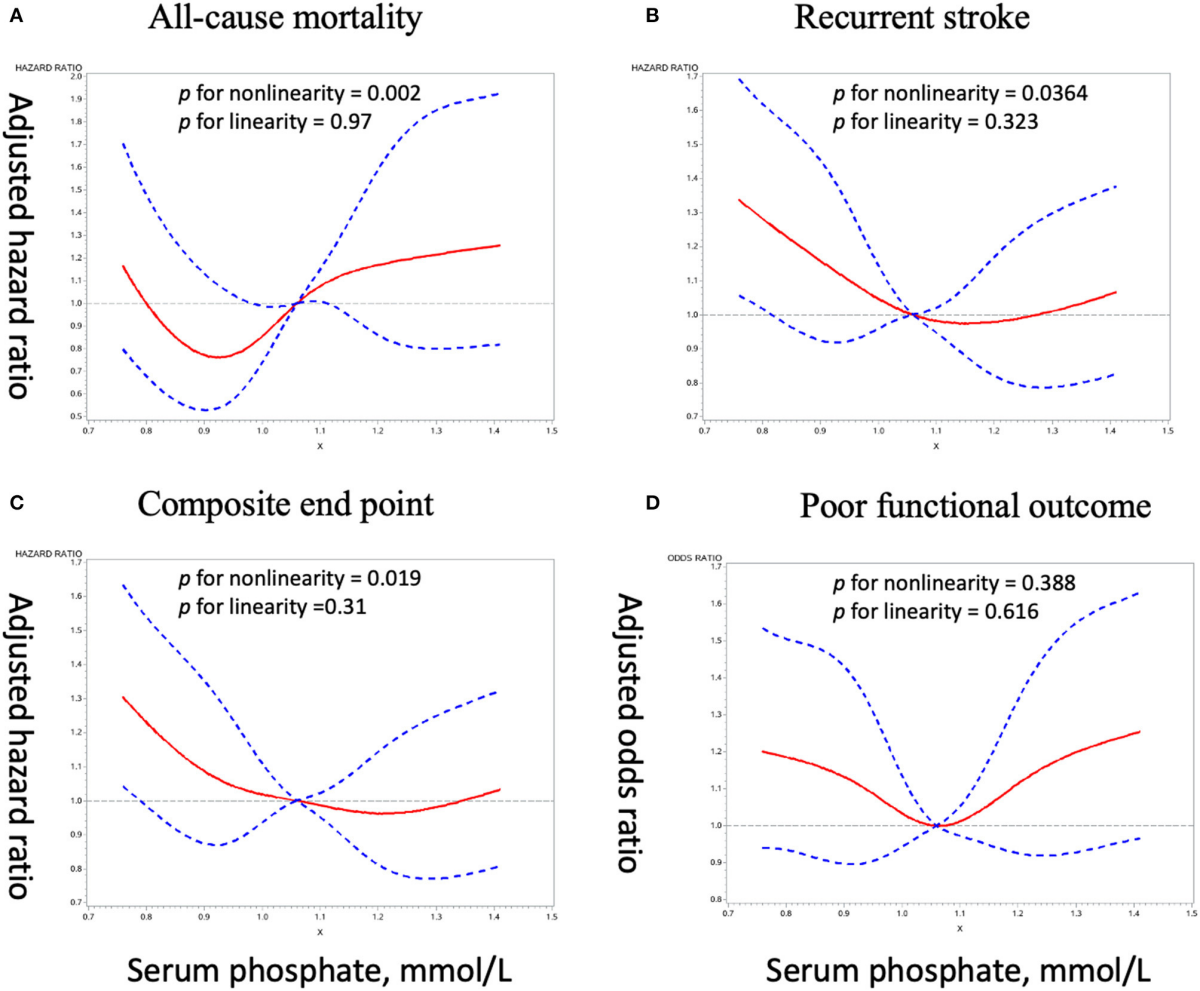
Adjusted dose–response association between serum phosphate levels and adverse clinical outcomes. **(A)**, All-cause mortality. **(B)**, Recurrent stroke. **(C)**, Composite end point. **(D)**, Poor functional outcome.

Crude and adjusted ORs or HRs with 95% CIs of serum phosphate levels for adverse clinical outcomes are presented in Table 3. Compared with the third quartile of phosphate (1.06–1.20 mmol/L), the adjusted ORs/ HRs (95% confidence interval) of the lowest quartile (<0.94 mmol/L) were 0.98 (0.67–1.42) for all-cause mortality, 1.31 (1.05–1.64) for stroke recurrence, 1.26 (1.02–1.57) for composite end point, and 1.27 (1.01–1.61) for poor functional outcome, and the adjusted ORs/HRs of the highest quartile (≥1.20 mmol/L) was 1.28 (0.87–1.86) for all-cause mortality, 1.11 (0.88–1.40) for stroke recurrence, 1.09 (0.87–1.35) for composite end point, and 1.40 (1.11–1.77) for poor functional outcome. Further analyses using restricted cubic spline regression showed a U-shaped relationship between serum phosphate levels and poor functional outcome and that low serum phosphate levels were significantly associated with increased risk of recurrent stroke and composite end point (Figure 2).

**Table 3:** Association between serum phosphate level and clinical outcomes.

	**Unadjusted**		**Age- and sex-adjusted**		**Multivariable-adjusted**^**†**^	
	**OR/HR (95% CI)**	***P*-value**	**OR/HR (95% CI)**	***P*-Value**	**OR/HR (95% CI)**	***P*-value**
**All-cause mortality**						
Q1 (<0.94 mmol/L)	1.41 (1.01-1.97)	0.04	1.17 (0.83-1.64)	0.37	0.98 (0.67-1.42)	0.90
Q2 (0.94-1.06 mmol/L)	0.93 (0.64-1.36)	0.72	0.83 (0.57-1.20)	0.32	0.78 (0.52-1.18)	0.24
Q3 (1.06-1.20 mmol/L)	1.00 (reference)		1.00 (reference)		1.00 (reference)	
Q4 (≥1.20 mmol/L)	1.25 (0.89-1.75)	0.21	1.32 (0.94-1.87)	0.11	1.28 (0.87-1.86)	0.21
**Recurrent stroke**						
Q1 (<0.94 mmol/L)	1.36 (1.10-1.67)	0.005	1.34 (1.08-1.66)	0.008	1.31 (1.05-1.64)	0.02
Q2 (0.94-1.06 mmol/L)	1.11 (0.89-1.39)	0.35	1.10 (0.88-1.38)	0.40	1.13 (0.90-1.43)	0.31
Q3 (1.06-1.20 mmol/L)	1.00 (reference)		1.00 (reference)		1.00 (reference)	
Q4 (≥1.20 mmol/L)	1.11 (0.89-1.38)	0.37	1.11 (0.89-1.38)	0.37	1.11 (0.88-1.40)	0.37
**Composite end point**						
Q1 (<0.94 mmol/L)	1.34 (1.09-1.64)	0.005	1.30 (1.06-1.60)	0.01	1.26 (1.02-1.57)	0.03
Q2 (0.94-1.06 mmol/L)	1.08 (0.87-1.34)	0.49	1.06 (0.85-1.32)	0.60	1.08 (0.87-1.36)	0.48
Q3 (1.06-1.20 mmol/L)	1.00 (reference)		1.00 (reference)		1.00 (reference)	
Q4 (≥1.20 mmol/L)	1.08 (0.88 −1.33)	0.48	1.09 (0.88-1.34)	0.44	1.09 (0.87-1.35)	0.46
**Poor functional outcome**						
Q1 (<0.94 mmol/L)	1.59 (1.32-1.91)	<0.0001	1.42 (1.17-1.72)	0.0003	1.27 (1.01-1.61)	0.04
Q2 (0.94-1.06 mmol/L)	1.33 (1.09-1.61)	0.004	1.24 (1.02-1.51)	0.03	1.24 (0.98-1.57)	0.07
Q3 (1.06-1.20 mmol/L)	1.00 (reference)		1.00 (reference)		1.00 (reference)	
Q4 (≥1.20 mmol/L)	1.30 (1.07-1.57)	0.007	1.36 (1.12-1.65)	0.002	1.40 (1.11-1.77)	0.005

† *In multivariable analysis, adjusted variables included age, sex, history of stroke, hypertension, diabetes mellitus, hypercholesterolemia, coronary heart disease, current or previous smoking, heavy alcohol, National Institutes of Health Stroke Scale score at admission, modified Rankin Scale score at discharge, body mass index, hemoglobin, serum calcium, serum potassium, serum albumin, estimated glomerular filtration rate, serum creatinine, total cholesterol, triglycerides, antihypertensive drugs, lipid-lowering drugs, hypoglycemia drugs, and pneumonia during hospitalization*.

*CI, confidence interval; HR, hazard ratios; OR, odds ratios; Q, quartiles*.

In patients with recurrent stroke (*n* = 674), 64 (9.5%) patients had hemorrhage stroke (Supplementary Table 2). Analyses for association between serum phosphate levels and 1-year hemorrhage stroke did not show statistical significance among groups (Supplementary Table 3).

Further analysis for association of serum phosphate levels with one-year all-cause mortality and poor functional outcome in patients without recurrent stroke (n = 6,679) did not show statistical difference between serum phosphate quartiles after adjusting for age, sex and other covariates. (Supplementary Table 4).

We further investigated whether adding serum phosphate to conventional risk factors improved the risk prediction of stroke recurrence, composite endpoint and poor functional outcome (Supplementary Table 5). Adding serum phosphate to a model containing conventional risk factors significantly improves risk reclassification for stroke recurrence (categorical net reclassification index was 8.9%, *p* = 0.03), composite end point (8.7%, *p* = 0.03) and poor functional outcome (14.3%, *p* < 0.001).

## Discussion

In this large observational study of patients with ischemic stroke and TIA, we found lower serum phosphate levels were associated with higher risk for stroke recurrence, composite end point and poor functional outcome after stroke, even after adjusting for potential covariates. Besides, higher serum phosphate levels were associated with higher risk for poor functional outcome. Our results provide new evidence for the relationship between serum phosphate levels and clinical outcomes after stroke.

Previous studies have demonstrated that both high and low serum phosphate levels could be associated with adverse cardiovascular events and related mortality (5–7, 9), and especially high serum phosphate received more attention (4, 23). However, the relationship between serum phosphate and adverse outcomes in patients with ischemic stroke has not been elucidated. Kim et al. (15) found that there is no association between serum phosphate levels and 3-month functional outcome in 1,034 patients with acute ischemic stroke. Zhong et al. (14) reported a U-shape relationship between serum phosphate and all-cause mortality in 2,944 acute ischemic stroke patients, and their results indicated that lower serum phosphate levels are associated with increased risk of in-hospital all-cause mortality. Our results also showed U-shaped relationships between serum phosphate and outcomes. We found that lower serum phosphate levels are associated with increased risk of 1-year stroke recurrence, composite end point and poor functional outcome, while higher serum phosphate levels are associated with increased risk of poor functional outcome. However, the association between serum phosphate levels and poor functional outcome lost significance when we further tested the relationship in patients without recurrent stroke. We speculated that the association between serum phosphate levels and poor functional outcome could be influenced by stroke recurrence since patients with recurrent stroke usually have poorer functional outcome (24).

The potential pathophysiological mechanisms about the association between low serum phosphate and adverse outcomes after stroke are unclear. There are several possible explanations. Lower serum phosphate levels may affect brain vascular biology considering phosphate as a component of cell membranes and it is important in mediating intracellular signaling (25). In addition, low serum phosphate is a manifestation of malnutrition and low physical activity (10–12). Therefore, further studies are warranted to determine whether the effect of serum phosphate on ischemic stroke outcomes is directly or it is just a manifestation of malnutrition in acute ischemic stroke patients. Furthermore, low phosphate was suggested to be related with hypertension, reduced insulin sensitivity, and metabolic syndrome (10, 26), which might help, although not directly, explain the relationship. More studies are needed to investigate the mechanism underlying the relationship between low phosphate and adverse outcomes. Previous studies considered the underlying pathological effects of elevated phosphate on cardiovascular organs involved vascular calcification and endothelial dysfunction (4, 27). Although restricting dietary phosphate intake was recommended for the benefit of cardiovascular interests (28), our results suggest that maintaining the serum phosphate within an appropriate level, instead of achieving a low phosphate level target, is important to help prevent stroke recurrence, composite end point and poor functional outcome. Further studies are needed to identify the most appropriate range of serum phosphate level.

The study had some limitations. First, this is an observational study. Although several important potential covariates had been adjusted in multivariable regression models, we could not rule out the possibility of residual confounding. For example, we could not adjust for pre-morbid nutrition status since our dataset did not measure pre-morbid function, nutrition intake or “frailty.” In addition, due to the fluctuation of serum phosphate with dietary intake, a single admission blood sample might not be representative of the serum phosphate profile over months and therefore interval samples depicting serum phosphate profiles are needed to clarify the relationship in the future study. Second, serum phosphate testing was performed at each study site. However, the results would be comparable because the same method based on the formation of ammonium phosphomolybdate using unfrozen samples was employed. Third, given that we excluded patients lacking serum phosphate value which partly depends on the clinical practice at individual participating hospitals, key covariate value and follow-up information, the selection bias might occur. Fourth, although the metabolism between serum phosphate and other serum electrolytes was closely related, due to that a large proportion of centers in our cohort did not test these variables such as serum magnesium routinely, we cannot further adjust for these variables. Finally, our study focused on Chinese population and the results might not be generalized to other populations with ischemic stroke.

## Conclusions

In summary, in patients with ischemic stroke and TIA, low serum phosphate levels were associated with increased risk of stroke recurrence, composite end point and poor functional outcome while high serum phosphate levels were associated with increased risk of poor functional outcome. Serum phosphate might serve as a predictor for stroke outcomes after ischemic stroke.

## Data Availability Statement

The raw data supporting the conclusions of this article will be made available by the authors, without undue reservation.

## Ethics Statement

The studies involving human participants were reviewed and approved by Ethics committee at Beijing Tiantan Hospital. The patients/participants provided their written informed consent to participate in this study.

## Author Contributions

J-FZ, JJ, XM, YP, Y-LW, X-QZ, J-XL, Y-CW, and Y-JW contributed to the conception and design of the study. J-FZ, JJ, XM, YP, Y-LW, X-QZ, J-XL, X-SH, B-BS, Z-CJ, S-DW, X-FC, W-JX, CA, Y-CW, and Y-JW contributed to the acquisition and analysis of data. J-FZ, JJ, XM, and YP contributed to drafting the text and preparing the figures. All authors edited and revised the manuscript and approved final submission.

## Conflict of Interest

The authors declare that the research was conducted in the absence of any commercial or financial relationships that could be construed as a potential conflict of interest.

## References

[1] Bringhurst F Demay B Krane S Kronenberg H. Bone and mineral metabolism in health and disease. In: Kasper D Braunwald E Fauci A Hauser S Longo D Jameson L, editors, et al., editors. Harrison's Principles of Internal Medicine. New York, NY: McGraw-Hill; (2004).

[2] Blumsohn A. What have we learnt about the regulation of phosphate metabolism? Curr Opin Nephrol Hypertens. (2004) 13:397–401. 10.1097/01.mnh.0000133983.40182.c3 15199289

[3] Fukagawa M Kurokawa K Papadakis M. Fluid and electrolyte disorders. In: Tierney L McPhee S Papadakis M, editors. Current Medical Diagnosis and Treatment 2005. New York, NY: McGraw-Hill/Appleton & Lange (2004).

[4] Ketteler M Wolf M Hahn K Ritz E. Phosphate: a novel cardiovascular risk factor. Eur Heart J. (2013) 34:1099–101. 10.1093/eurheartj/ehs247 23045267

[5] Dhingra R Sullivan LM Fox CS Wang TJ D'Agostino RB Sr. Gaziano JM. Relations of serum phosphorus and calcium levels to the incidence of cardiovascular disease in the community. Arch Intern Med. (2007) 167:879–85. 10.1001/archinte.167.9.879 17502528

[6] Foley RN Collins AJ Ishani A Kalra PA. Calcium-phosphate levels and cardiovascular disease in community-dwelling adults: the Atherosclerosis Risk in Communities (ARIC) Study. Am Heart J. (2008) 156:556–63. 10.1016/j.ahj.2008.05.016 18760141

[7] Dominguez JR Kestenbaum B Chonchol M Block G Laughlin GA Lewis CE. Relationships between serum and urine phosphorus with all-cause and cardiovascular mortality: the Osteoporotic Fractures in Men (MrOS) Study. Am J Kidney Dis. (2013) 61:555–63. 10.1053/j.ajkd.2012.11.033 23261120 PMC3815620

[8] Dhingra R Gona P Benjamin EJ Wang TJ Aragam J D'Agostino RB Sr.. Relations of serum phosphorus levels to echocardiographic left ventricular mass and incidence of heart failure in the community. Eur J Heart Fail. (2010) 12:812–8. 10.1093/eurjhf/hfq106 20675668 PMC2913049

[9] Tonelli M Sacks F Pfeffer M Gao Z Curhan G. Relation between serum phosphate level and cardiovascular event rate in people with coronary disease. Circulation. (2005) 112:2627–33. 10.1161/circulationaha.105.553198 16246962

[10] DeFronzo RA Lang R. Hypophosphatemia and glucose intolerance: evidence for tissue insensitivity to insulin. N Engl J Med. (1980) 303:1259–63. 10.1056/NEJM198011273032203 6999353

[11] Ljunghall S Hedstrand H. Serum phosphate inversely related to blood pressure. Br Med J. (1977) 1:553–4. 10.1136/bmj.1.6060.553 843804 PMC1605172

[12] Park W Kim BS Lee JE Huh JK Kim BJ Sung KC. Serum phosphate levels and the risk of cardiovascular disease and metabolic syndrome: a double-edged sword. Diabetes Res Clin Pract. (2009) 83:119–25. 10.1016/j.diabres.2008.08.018 19101054

[13] Block GA Hulbert-Shearon TE Levin NW Port FK. Association of serum phosphorus and calcium x phosphate product with mortality risk in chronic hemodialysis patients: a national study. Am J Kidney Dis. (1998) 31:607–17. 10.1053/ajkd.1998.v31.pm9531176 9531176

[14] Zhong C You S Chen J Zhai G Du H Luo Y. Serum Alkaline Phosphatase, Phosphate, and In-Hospital Mortality in Acute Ischemic Stroke Patients. J Stroke Cerebrovasc Dis. (2018) 27:257–66. 10.1016/j.jstrokecerebrovasdis.2017.08.041 28986200

[15] Kim J Song TJ Song D Lee HS Nam CM Nam HS. Serum alkaline phosphatase and phosphate in cerebral atherosclerosis and functional outcomes after cerebral infarction. Stroke. (2013) 44:3547–9. 10.1161/strokeaha.113.002959 24021686

[16] Wang Y Jing J Meng X Pan Y Wang Y Zhao X. The Third China National Stroke Registry (CNSR-III) for patients with acute ischaemic stroke or transient ischaemic attack: design, rationale and baseline patient characteristics. Stroke Vasc Neurol. (2019) 4:158–64. 10.1136/svn-2019-000242 31709123 PMC6812638

[17] Pearson TA Palaniappan LP Artinian NT Carnethon MR Criqui MH Daniels SR. American Heart Association Guide for Improving Cardiovascular Health at the Community Level, 2013 update: a scientific statement for public health practitioners, healthcare providers, and health policy makers. Circulation. (2013) 127:1730–53. 10.1161/CIR.0b013e31828f8a94 23519758

[18] American Diabetes Association. Classification and Diagnosis of Diabetes: Standards of Medical Care in Diabetes-2020. Diabetes Care. (2020) 43:S14-s31. 10.2337/dc20-S002 31862745

[19] Joint committee for guideline revision. 2016 Chinese guidelines for the management of dyslipidemia in adults. J Geriatr Cardiol. (2018) 15:1–29. 10.11909/j.issn.1671-5411.2018.01.011 29434622 PMC5803534

[20] Teo BW Xu H Wang D Li J Sinha AK Shuter B. GFR estimating equations in a multiethnic Asian population. Am J Kidney Dis. (2011) 58:56–63. 10.1053/j.ajkd.2011.02.393 21601325

[21] Wang X Luo Y Wang Y Wang C Zhao X Wang D. Comparison of associations of outcomes after stroke with estimated GFR using Chinese modifications of the MDRD study and CKD-EPI creatinine equations: results from the China National Stroke Registry. Am J Kidney Dis. (2014) 63:59–67. 10.1053/j.ajkd.2013.08.008 24100127

[22] Pencina MJ D'Agostino RB Sr. D'Agostino RB Jr. Vasan RS. Evaluating the added predictive ability of a new marker: from area under the ROC curve to reclassification and beyond. Stat Med. (2008) 27:157–72; discussion 207–12. 10.1002/sim.2929 17569110

[23] Chang AR Lazo M Appel LJ Gutiérrez OM Grams ME. High dietary phosphorus intake is associated with all-cause mortality: results from NHANES III. Am J Clin Nutr. (2014) 99:320–7. 10.3945/ajcn.113.073148 24225358 PMC3893724

[24] Wang A Wu L Wang X Zhao X Wang C Liu L. Effect of recurrent stroke on poor functional outcome in transient ischemic attack or minor stroke. Int J Stroke. (2016) 11:Np80. 10.1177/1747493016641954 27012273

[25] Yamada S Tsuruya K Taniguchi M Tokumoto M Fujisaki K Hirakata H. Association between serum phosphate levels and stroke risk in patients undergoing hemodialysis: the Q-cohort study. Stroke. (2016) 47:2189–96. 10.1161/strokeaha.116.013195 27507862

[26] Kalaitzidis R Tsimihodimos V Bairaktari E Siamopoulos KC Elisaf M. Disturbances of phosphate metabolism: another feature of metabolic syndrome. Am J Kidney Dis. (2005) 45:851–8. 10.1053/j.ajkd.2005.01.005 15861350

[27] Heine GH Nangaku M Fliser D. Calcium and phosphate impact cardiovascular risk. Eur Heart J. (2013) 34:1112–21. 10.1093/eurheartj/ehs353 23109644

[28] Scialla JJ Wolf M. Roles of phosphate and fibroblast growth factor 23 in cardiovascular disease. Nat Rev Nephrol. (2014) 10:268–78. 10.1038/nrneph.2014.49 24686452

