# Supplementary material for: Serum Phosphate and 1-Year Outcome in Patients With Acute Ischemic Stroke and Transient Ischemic Attack
Source: Front Neurol. 2021 Apr 14;12:652941. doi: 10.3389/fneur.2021.652941 (PMC8079723; doi:10.3389/fneur.2021.652941)
Supplement: Supplementary file 1 [file Table_1.DOCX]

**SUPPLEMENTARY MATERIAL**

SUPPLEMENTARY Table I. The characteristics of the excluded and included patients

| Characteristics | Excluded (n=7813) | Included (n=7353) | P value |
| --- | --- | --- | --- |
| Age (mean ± SD), y | 62.0±11.2 | 62.5±11.4 | 0.004 |
| Male sex, n (%) | 5318 (68.1) | 5046 (68.6) | 0.46 |
| Risk factors, n (%) | | | |
| Previous stroke | 1778 (22.8) | 1577 (21.5) | 0.05 |
| Hypertension | 4847 (62.0) | 4647 (63.2) | 0.14 |
| Diabetes mellitus | 1746 (22.4) | 1764 (24.0) | 0.02 |
| Hypercholesterolemia | 602 (7.7) | 589 (8.0) | 0.49 |
| CHD | 831 (10.6) | 777 (10.6) | 0.89 |
| Current or previous smoking | 3806 (48.7) | 3514 (47.8) | 0.26 |
| Heavy drinker | 1042 (13.3) | 1084 (14.7) | 0.01 |
| BMI (mean±SD) | 24.7±3.3 | 24.7±3.4 | 0.32 |
| NIHSS score at admission, median (IQR) | 3 (1-6) | 3 (1-6) | 0.05 |
| mRS score at discharge, n (%) | | | |
| 0-2 | 6487 (83.1) | 5977 (81.6) | 0.01 |
| 3-5 | 1316 (16.9) | 1351 (18.4) |  |

Abbreviations: BMI, body mass index; CHD, coronary heart disease; IQR, interquartile range; mRS, modified Rankin Scale score; NIHSS, National Institutes of Health Stroke Scale; Q, Quartiles.

SUPPLEMENTARY Table II. Rates of one-year hemorrhage stroke according to quartiles of serum phosphate level

|  |  | Serum Phosphate Level, mmol/L | | | |  |
| --- | --- | --- | --- | --- | --- | --- |
| Outcomes | Overall | Q1(<0.94) | Q2(0.94-1.06) | Q3(1.06-1.20) | Q4(≥1.20) | p value |
| Hemorrhage stroke, n (%) | 64 (0.87) | 20 (1.09) | 13 (0.75) | 14 (0.74) | 17 (0.90) | 0.62 |

Q, Quartiles.

SUPPLEMENTARY Table III. Association between serum phosphate level and one-year hemorrhage stroke

|  | Unadjusted | | Age- and Sex-Adjusted | | Multivariable  - Adjusted † | |
| --- | --- | --- | --- | --- | --- | --- |
|  | OR/HR (95% CI) | P Value | OR/HR (95% CI) | P Value | OR/HR (95% CI) | P Value |
| Hemorrhage stroke | | | | | | |
| Q1 (<0.94 mmol/L) | 1.50 (0.76-2.96) | 0.25 | 1.46 (0.73-2.92) | 0.28 | 1.52 (0.73-3.16) | 0.26 |
| Q2 (0.94-1.06 mmol/L) | 1.02 (0.48-2.17) | 0.96 | 1.01 (0.47-2.15) | 0.97 | 0.90 (0.40-2.03) | 0.80 |
| Q3 (1.06-1.20 mmol/L) | 1.00 (reference) |  | 1.00 (reference) |  | 1.00 (reference) |  |
| Q4 (≥1.20 mmol/L) | 1.23 (0.61-2.49) | 0.57 | 1.25 (0.61-2.54) | 0.54 | 1.32 (0.63-2.73) | 0.46 |

† In multivariable analysis, adjusted variables included age, sex, history of stroke, hypertension, diabetes mellitus, hypercholesterolemia, coronary heart disease, current or previous smoking, heavy alcohol, baseline National Institutes of Health Stroke Scale score, modified Rankin Scale score at discharge, body mass index, hemoglobin, serum calcium, serum potassium, serum albumin, estimated glomerular filtration rate, serum creatinine, total cholesterol, triglycerides, antihypertensive drugs, lipid-lowering drugs, hypoglycemia drugs, and pneumonia during hospitalization.

Abbreviation: CI, confidence interval; HR, hazard ratios; OR, odds ratios; Q, Quartiles.

SUPPLEMENTARY Table IV. Association between serum phosphate level and one-year all-cause mortality and poor functional outcome in patients without recurrent stroke (n=6679)

|  | Unadjusted | | Age- and Sex-Adjusted | | Multivariable  - Adjusted † | |
| --- | --- | --- | --- | --- | --- | --- |
|  | OR/HR (95% CI) | P Value | OR/HR (95% CI) | P Value | OR/HR (95% CI) | P Value |
| All-cause mortality | | | | | | |
| Q1 (<0.94 mmol/L) | 1.53 (1.02-2.28) | 0.04 | 1.20 (0.80-1.80) | 0.38 | 0.96 (0.61-1.52) | 0.86 |
| Q2 (0.94-1.06 mmol/L) | 0.89 (0.56-1.41) | 0.63 | 0.77 (0.49-1.22) | 0.26 | 0.68 (0.41-1.13) | 0.14 |
| Q3 (1.06-1.20 mmol/L) | 1.00 (reference) |  | 1.00 (reference) |  | 1.00 (reference) |  |
| Q4 (≥1.20 mmol/L) | 1.24 (0.82-1.88) | 0.30 | 1.34 (0.89-2.04) | 0.16 | 1.22 (0.77-1.95) | 0.39 |
| Poor functional outcome | | | | | | |
| Q1 (<0.94 mmol/L) | 1.58 (1.28-1.95) | <0.0001 | 1.41 (1.13-1.75) | 0.002 | 1.22 (0.93-1.59) | 0.15 |
| Q2 (0.94-1.06 mmol/L) | 1.25 (1.01-1.56) | 0.045 | 1.16 (0.93-1.46) | 0.19 | 1.12 (0.86-1.47) | 0.40 |
| Q3 (1.06-1.20 mmol/L) | 1.00 (reference) |  | 1.00 (reference) |  | 1.00 (reference) |  |
| Q4 (≥1.20 mmol/L) | 1.23 (0.99-1.52) | 0.06 | 1.27 (1.02-1.59) | 0.03 | 1.30 (0.99-1.69) | 0.06 |

† In multivariable analysis, adjusted variables included age, sex, history of stroke, hypertension, diabetes mellitus, hypercholesterolemia, coronary heart disease, current or previous smoking, heavy alcohol, baseline National Institutes of Health Stroke Scale score, modified Rankin Scale score at discharge, body mass index, hemoglobin, serum calcium, serum potassium, serum albumin, estimated glomerular filtration rate, serum creatinine, total cholesterol, triglycerides, antihypertensive drugs, lipid-lowering drugs, hypoglycemia drugs, and pneumonia during hospitalization.

Abbreviation: CI, confidence interval; HR, hazard ratios; OR, odds ratios; Q, Quartiles.

SUPPLEMENTARY Table V. Reclassification and discrimination statistics for one-year clinical outcomes by serum phosphate among patients with acute ischemic stroke

| model | C statistic | | NRI (categorical) | | IDI | |
| --- | --- | --- | --- | --- | --- | --- |
|  | Estimate (95% CI) | p Value | Estimate (95% CI), % | p Value | Estimate (95% CI), % | p Value |
| **Stroke recurrence** |  |  |  |  |  |  |
| Conventional model | 0.594(0.571-0.617) |  | Reference |  | Reference |  |
| Conventional model + serum phosphate | 0.599(0.576-0.622) | 0.22 | 8.9(1.2-16.6) | 0.03 | 0.09(0.01-0.17) | 0.02 |
| **Composite end point** |  |  |  |  |  |  |
| Conventional model | 0.603(0.580-0.625) |  | Reference |  | Reference |  |
| Conventional model + serum phosphate | 0.606(0.584-0.628) | 0.22 | 8.7 (1.4-16.0) | 0.03 | 0.08(0.01-0.16) | 0.03 |
| **Poor functional outcome** |  |  |  |  |  |  |
| Conventional model | 0.793(0.779-0.808) |  | Reference |  | Reference |  |
| Conventional model + serum phosphate | 0.795(0.781-0.810) | 0.06 | 14.3(8.1-20.6) | <0.001 | 0.13(0.01-0.25) | 0.04 |
|  |  |  |  |  |  |  |

Conventional model included age, sex, history of stroke, hypertension, diabetes mellitus, dyslipidemia, coronary heart disease, smoking, heavy drinker and baseline National Institutes of Health Stroke Scale score.

Abbreviations: CI, confidence interval; NRI, net reclassification index; IDI, integrated discrimination improvement.
